# Evolution of Salvage Radical Prostatectomy from Open to Robotic and Further to Retzius Sparing Surgery

**DOI:** 10.3390/jcm11010202

**Published:** 2021-12-30

**Authors:** Viktoria Schuetz, Philipp Reimold, Magdalena Goertz, Luisa Hofer, Svenja Dieffenbacher, Joanne Nyarangi-Dix, Stefan Duensing, Markus Hohenfellner, Gencay Hatiboglu

**Affiliations:** 1 Department of Urology, University Hospital Heidelberg, 69120 Heidelberg, Germany; philipp.reimold@med.uni-heidelberg.de (P.R.); magdalena.goertz@med.uni-heidelberg.de (M.G.); Luisa.Hofer@med.uni-heidelberg.de (L.H.); svenja.dieffenbacher@med.uni-heidelberg.de (S.D.); joan.nyarangi-dix@med.uni-heidelberg.de (J.N.-D.); stefan.duensing@med.uni-heidelberg.de (S.D.); Hohenfellner@med.uni-heidelberg.de (M.H.); 2 Section of Molecular Urooncology, Department of Urology, Medical Faculty Heidelberg, University Hospital Heidelberg, 69120 Heidelberg, Germany

**Keywords:** salvage prostatectomy, open radical prostatectomy, robotic radical prostatectomy, Retzius-sparing

## Abstract

Salvage radical prostatectomy (sRP) has evolved from open to minimally invasive approaches. sRP can be offered to patients with local recurrence to improve biochemical recurrence (BCR)-free and overall survival. We evaluate oncological outcome and continence after retropubic (RRP), conventional (cRARP), and Retzius-sparing robotic (rsRARP) surgery. Materials/methods: A total of 53 patients undergoing sRP between 2010 and 2020 were included. Follow-up included oncological outcome and continence. Results: sRP was done as RRP (*n* = 25), cRARP (*n* = 7), or rsRARP (*n* = 21). Median blood loss was 900 mL, 500 mL, and 300 mL for RRP, cRARP, and rsRARP, respectively. At 12 months, 5 (20%), 0, and 4 (19%) patients were continent, 9 (36%), 3 (43%), and 7 (33%) had grade 1 incontinence, 5 (20%), 2 (29%), and 3 (14%) had grade 2 incontinence, and 3 (12%), 2 (29%), and 4 (19%) had grade 3 incontinence for RRP, cRARP, or rsRARP, respectively. During a mean follow-up of 52.6 months, 16 (64%), 4 (57%), and 3 (14%) developed BCR in the RRP-, cRARP-, and rsRARP-group, respectively. Conclusions: Over the years, sRP has shifted from open to laparoscopic/robotic surgery. RARP shows good oncological and functional outcome. rsRARP ensures direct vision on the rectum during preparation and can therefore increase safety and surgeon’s confidence, especially in the salvage setting.

## 1. Introduction

Prostate cancer (PCa) is the most common non-cutaneous cancer in men. The standard treatment for local prostate cancer includes radical prostatectomy (RP) or primary radiation treatment (RT) [1]. Approximately 27 to 53% of patients will develop biochemical recurrence (BCR) after RP or RT [1]. Other experimental treatment options such as thermoablation (e.g., HIFU) have even higher rates of local recurrence [2]. For patients with histological proven local recurrence after primary RT or other organ preserving local treatments, salvage radical prostatectomy (sRP) provides a curative option in well-selected cases [3] with intermediate term good cancer control [4] leading to BCR-free survival at 5 and 10 years of 47–82% and 28–53%, respectively [3]. However, sRP is not offered to all patients with regard to historic series due to high rates of procedure-related side effects [5]. Instead, these patients receive antihormonal, palliative treatment, missing the opportunity for a potential cure of their disease. At five years after sRP, recent publications show a BCR, CSS, and OS of 56.7%, 97.7%, and 92.1%, respectively [4]. Salvage prostatectomy can be done using different approaches. Retropubic radical prostatectomy (RRP) as described by Walsh et al. has been the standard surgical approach for decades [6]. However, minimally invasive techniques, especially robotic radical prostatectomy (RARP), have widely replaced open surgery in the primary setting [7]. The advantages of this minimally invasive technique have been well documented and described for primary treatment of localized prostate cancer. For the non-salvage situation, level 1 evidence demonstrates that RARP is associated with lower blood loss, a shorter hospital stay, and shorter operation time than RRP [8]. Regarding functional and oncological outcomes, studies also demonstrated at least similar results for both techniques [8,9]. Early reports described retropubic radical prostatectomy as a salvage treatment in selected patients with local recurrence after primary irradiation treatment [10]. In contrast to primary surgery, sRP is associated with a higher risk for urinary incontinence as well as other complications such as rectal injury or anastomotic stricture [11]. Erectile dysfunction will occur in nearly all patients after sRP [3]. With the evolution from open to robotic surgery, first studies have also evaluated robotic sRP and demonstrated its safety and feasibility [12,13]. In comparison to open sRP, safety and outcome are similar [14] with salvage RARP having lower blood loss and shorter hospital stay [15].

Conventional robotic radical prostatectomy (cRARP) mimics the approach of open surgery with anterior dissection of the prostate via the Retzius space [16] and is still the most widely used approach for primary treatment as an online poll recently demonstrated [17]. Bocciardi et al. developed a new technique for prostatectomy in 2010 that is exclusive for the robot and cannot be performed by open surgery [18]. Hereby the dissection of the prostate and bladder neck is being done in a dorsal, pararectal approach via the recto-vesical pouch, leaving the Retzius space and the attachments of the bladder and the urethra untouched (Retzius-sparing RARP). Several studies so far have demonstrated a better postoperative continence using this approach [17,18,19,20]. This approach has never been evaluated for sRP. The purpose of this manuscript was to demonstrate the steps of evolution from open to RARP with the dorsal approach as a possible next step of evolution in the salvage setting.

## 2. Patients and Methods

### 2.1. Study Population

All patients undergoing salvage radical prostatectomy for recurrent prostate cancer after primary local therapy (radiotherapy (*n* = 37), HIFU (*n* = 14), irreversible electroporation (*n* = 1), cryoablation (*n* = 1)) from our institution between 2010 and 2020 were included. Confirmatory biopsies were performed before salvage treatment. All patients gave written consent. The study protocol was approved by the local ethics committee (S-335/2021). The presented data highlights the evolution from open surgery to cRARP and further to Retzius-sparing RARP (rsRARP) for prostatectomy at our institution. Therefore, patients were treated according to the best surgical approach at time of surgery.

### 2.2. Collected Parameters

The parameters collected included the following preoperative variables: patient age at time of diagnosis and operation, body mass index, initial Gleason-score, PSA level and clinical stage, prior local treatment, Gleason-score, PSA level, clinical stage, prostate volume, and Digital rectal examination (DRE) at recurrence. The peri- and postoperative parameters collected included surgical approach, operation time, blood loss, number of blood transfusions, bladder-neck preservation, nerve-sparing, intra-operative water tightness of the anastomosis, final pathological T stage, surgical margins, peri-operative androgen deprivation treatment (ADT), length of hospital stay, duration of Foley catheter, post-operative complications, and immediate continence. Margin status was classified as <3 mm, ≥3 mm, or multiple (regardless of length). Clinically significant positive surgical margin (PSM) was defined as ≥3 mm or as multiple [21]. All patients were followed up every three months including continence rates, PSA values, time to recurrence, and overall survival. Incontinence was graded as follows: continent (patients without significant urine loss and using a maximum of one security pad/day), grade 1 (urine loss during heavy physical activity such as coughing, sneezing, pressure, and laughing), grade 2 (urine loss during moderate physical activity such as lifting, walking, running, and climbing stairs), grade 3 (urine loss during standing without physical activity).

### 2.3. Surgical Approach for Salvage Radical Prostatectomy

RRP was performed as retropubic, ascending approach. RARP was done transperitoneal using a DaVinci si system (Intuitive surgical, Sunnyvale, CA, USA) either by Montsouri’s technique (cRARP) [16] or using the Retzius sparing approach (rsRARP) as described by Bocciardi et al. [18]. In comparison to Montsouri’s approach, the latter technique leaves the bladder attached to the abdominal wall during the entire preparation of the prostate. Surgery is done via a pararectal incision in the recto-vesical pouch [18]. This allows preparation in line of sight with a permanent view onto the rectum. Nerve sparing was not performed in most cases for oncological safety as well as feasibility due to fibrosis of the tissue due to previous treatment. The anastomosis was done with 6–8 intermittent sutures in RRP and running suture in robotic approach, using two monofilic filaments for the anterior and posterior wall of the anastomosis [22] and later on with barbed sutures. Water tightness was tested intraoperatively [23]. A 20 French Foley catheter was placed. A suprapubic catheter was placed in some cases. Extended lymph node dissection was routinely performed.

### 2.4. Follow-Up

All patients received their first follow-up directly after catheter removal, evaluating immediate continence. For further follow-up stress-incontinence, pad-use per day and PSA-level were noted every three months by patient interview.

### 2.5. Statistical Analysis

All patients and data available were included in the analysis. Descriptive analysis was given by median, range, mean, and standard deviation (SD) for the above-mentioned variables. Patients were grouped according to the surgical approach. Comparison between groups was done by Kruskal–Wallis for continuous variables and Chi-square for categorical variables. Kaplan–Meier curves were constructed for time to biochemical recurrence. All statistical analysis was carried out using IBM SPSS software version 26.

## 3. Results

A total of 53 patients underwent sRP between 2010 and 2020 for recurrent prostate cancer (25 patients (47.2%) RRP; 28 patients (52.8%) RARP). Among RARP patients, cRARP technique was performed in 7 cases (13.2%) while rsRARP was done in 21 (39.6%) men. The mean age including all patients at the time of surgery was 67.6 (±5.9) years. Salvage prostatectomy was performed for local recurrence after primary external beam radiation therapy in 27 patients (50.9%), C12 ion irradiation therapy in 3 patients (5.7%), HIFU therapy in 14 patients (26.4%), HDR and LDR brachytherapy (1 patient (1.9%) and 6 patients (11.3%), respectively), and IRE and cryoablation in 1 patient (1.9%) each. In total, 5 patients already received salvage therapy for local recurrence (salvage HIFU in 4 patients after radiotherapy and salvage radiotherapy for 1 patient after HIFU). In these patients, salvage prostatectomy was the second salvage therapy performed. Metastatic disease was ruled out in all patients by radiographic studies. Mean time until recurrence after previous therapy was 52.8 (±33.5) months (therefore, some initial pathology results are given in the former Gleason grading system used before 2005 including Gleason score ≤ 5). On recurrence, 30 patients (56.6%) presented with a clinical stage cT2, 8 patients (15.1%) with a stage cT3, and one patient with a non-organ confined stage cT4. Fourteen patients (26.4%) received androgen deprivation therapy (ADT) prior to surgery. Patient characteristics between surgical approaches was not equally distributed in terms of initial PSA and PSA before salvage treatment as shown in Table 1. Complete perioperative data were available for all patients and are given as descriptive data in Table 2.

Median operation time for sRP was 205 (IQR: 176–225) mins, 252 (IQR: 210–330) mins and 228 (IQR: 206–259) mins, while median blood loss was 900 mL (IQR: 550–1350), 500 mL (IQR: 300–600), and 300 mL (IQR 200–500) for RRP, cRARP, and rsRARP, respectively. Hospital stay was median 5 days in both RARP groups and 9 days in the RRP group. Indwelling catheter time was 20 (13–34) d, 27 (20–96) d, and 37 (27–51.5) d after rsRARP, cRARP, and RRP, respectively. Rectal injury occurred in one patient in the RRP group. No further perioperative complications were recorded. Statistically significant differences between robotic surgery (cRARP and rsRARP) vs. open surgery were seen for blood loss (*p* < 0.01), hospital stay (*p* < 0.01), catherization (*p* = 0.02), and operation time (*p* = 0.01). No differences were noted in comparison of both robotic techniques.

Immediately after catheter removal, continence was reported by 14.3% (*n* = 3) after rsRARP and 8.0% (*n* = 2) after RRP. Grade I stress incontinence was present in 7 (28.0%), 1 (14.3%), and 3 (14.3%); grade II 8 (32.0%), 4 (57.1%), and 4 (19.0%); and grade III stress incontinence in 8 (32.0%), 1 (14.3%), and 5 (23.8%) patients after RRP, cRARP, and rsRARP, respectively. One year after surgery, continence was reported by 5 patients (20.0%), none, and 4 patients (19.0%) after RRP, cRARP, and rsRARP, respectively. Stress incontinence Grade I was present in 9 (36.0%), 3 (42.9%), and 7 (33.3%); Grade II in 5 (20.0%), 2 (28.6%), and 3 (14.3%); and Grade III in 3 (12.0%), 2 (28.6%), and 4 (19.0%) patients for RRP, cRARP, and rsRARP, respectively. No statistical differences could be seen.

Overall, 62.3% (*n* = 33) had negative surgical margins (RRP *n* = 15 (60.0%), cRARP *n* = 2 (28.6%), rsRARP *n* = 16 (76.2%)), positive surgical margins (PSM) were present in 32.1% (*n* = 17). One resection (1.9%) was incomplete (R2) and surgical margins were reported by the pathologist as “RX” for two patients (3.8%). Positive surgical margins were further analyzed by location and clinical significance. Patients with localized prostate cancer (pT2) had any PSM in 2/25 (8%), 0/7 (0%), and 2/21 (9.5%) and clinically significant PSM in 1/25 (4%), 0/7 (0%), and 1/21 (4.8%), while patients with advanced prostate cancer (for ≥pT3) had any PSM in 8/25 (32%), 4/7 (57%), and 2/21 (9.5%) and clinically significant PSM in 8/25 (32%), 4/7 (57%), and 1/21 (4.8%) for RRP, cRARP, and rsRARP, respectively. Localization of PSM for clinically significant tumor was apical (*n* = 1, pT2), bladder neck (*n* = 1, pT3), lateral (*n* = 1, pT3), seminal vesical (*n* = 1, pT4), and multifocal (*n* = 5, pT3) for RRP, multifocal (*n* = 3, pT3) and ductus deferens (*n* = 1, pT3) for cRARP, and multifocal (*n* = 2 pT2 and pT3) for rsRARP. No significant differences could be seen comparing all study groups or open and robotic surgery (*p* > 0.05) while PSM where lower in rsRARP compared to cRARP (*p* = 0.03).

**Figure 1:**
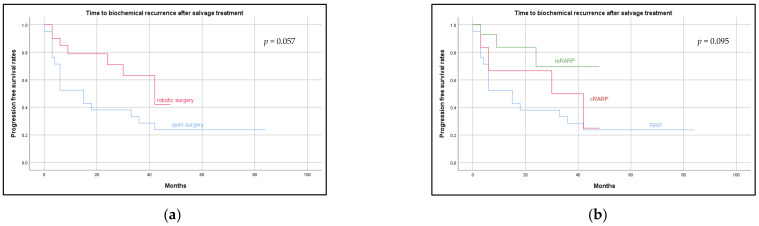
Time to biochemical recurrence. (**a**) Showing time to biochemical recurrence after salvage prostatectomy for open vs. robotic assisted radical prostatectomy. (**b**) Showing time to biochemical recurrence separately for open (RRP), conventional (cRARP), and Retzius-sparing (rsRARP) robotic salvage prostatectomy.

Biochemical recurrence (BCR) occurred in 16 (64.0%), 4 (57.1%), and 3 (14.3%) patients after RRP, cRARP, and rsRARP, respectively. Median follow-up for all patients was 30 months (IQR: 9.0–45.0), for RRP 42 months (IQR: 22.5–66.0), for cRARP 36 months (IQR: 30.0–48.0), and for rsRARP 12 months (IQR: 6.0–27.0). Average time to recurrence was 12.4 (IQR: 3.0–17.3) months, 20.3 (IQR: 3.8–39.0) months, and 12.0 (ICQ: not applicable) months after RRP, cRARP, and rsRARP, respectively. Time until recurrence is presented below as Kaplan–Maier plots (Figure 1). During follow-up, 8 patients died.

## 4. Discussion

We report our experience and results of salvage prostatectomy over the past ten years presenting the evolution from open to minimally invasive robotic, Retzius-sparing approach evaluating a total of 53 patients undergoing sRP with different approaches (RRP, cRARP, rsRARP) and with regard to perioperative side effects, continence, and oncological results.

Currently, minimally invasive radical prostatectomy is the gold standard procedure for the surgical treatment of localized prostate cancer, providing excellent oncological results with minimal side effects [24]. However, the procedure has undergone a long evolution to become the safe procedure that it represents today. Initial experiences described in 1947 for retropubic approach [25] did not gain popularity because of extensive blood loss and major side effects on functional outcome resulting from a lack of knowledge of peri-prostatic anatomy. The change came with studies by Walsh et al. presenting the technique of anatomical radical prostatectomy leading to a significant reduction in morbidity [6,26,27]. With the introduction of laparoscopic and robotic surgery, a new era in radical prostatectomy began and has since become the standard of care in the primary setting [17]. Hereby the surgical technique was adopted directly from open surgery to RARP. Retzius-sparing RARP was introduced in 2010 [18] as a technique exclusively for robotic surgery and therefore taking full advantage of the robotic system. So far, only a few studies have been published describing robot-assisted sRP, none of them describing rsRARP for salvage surgery.

The challenge in salvage surgery is the preservation of functional outcome without compromising oncological results. Salvage surgery is more demanding due to a higher risk for side effects such as incontinence, erectile dysfunction, and rectal injuries. After primary radiation treatment peri-prostatic anatomy is altered due to tissue adhesions, radiation-induced inflammation, and fibrosis [28]. Historical series for sRP showed a risk for rectal injury of up to 35%. Additionally, incontinence rates up to 73% are described [29]. Because of these disappointing results, only a few patients were offered or willing to undergo local salvage treatment [30,31].

With regards to the challenging procedure, sRP was a domain of open surgery and mainly done in high volume centers. Compared to historical series, recent publications demonstrate better functional outcome. However, incontinence rate is still higher than in the primary setting. Gontero et al. describe continence rates for RRP of 22.5% and mild incontinence in 35% after 12 months [15]. These results were therefore comparable to the current series.

As a matter of fact, the evolution of radical prostatectomy towards robotic surgery was adopted to the salvage setting. Different authors describe the feasibility and compare functional and oncological outcome. Thereby the authors postulate better continence rates and less surgical morbidity in the minimally invasive setting [13,15,32,33]. In our series, the robotic approach was also associated with lower blood loss, shorter operation time, and hospital stay as well as shorter catheter indwelling time. However, a direct comparison of both techniques in the retrospective setting must be considered with care as they evolved sequentially over time. Reliable results need prospective randomized evaluation.

With regard to other publications, robotic surgery is usually done as cRARP, a technique that translates open surgery directly to the robotic setting. As a further step in surgical evolution, we describe the Retzius-sparing procedure in the salvage setting. Major advantages of this approach are the preparation with direct vision on Denonvilliers’ fascia and the anterior rectal wall. Although cRARP has an initial dorsal approach with the release of the prostate from the anterior rectal wall, major parts of the surgery—especially preparation of the apical regions and dissection of the vascular pedicels—are done from the anterior [16]. As rectal injuries are of major concern for the salvage procedure, rsRARP can increase patient safety and surgeons’ confidence. In our opinion, complete dorsal preparation in line of sight with a permanent view onto the rectum is an advantage and reassurance during surgery. However, no rectal injury occurred in our series for either technique of robotic surgery, demonstrating the safety of both techniques. Our results correlate with current literature [13,14,34]. In comparison, historic series for open salvage prostatectomy report of rectal injuries in up to 19% [35].

While our data showed advantages for the robotic approach, a comparison of cRARP with rsRARP has to be considered with care, as reliable results would need larger patient cohorts and ideally a randomization of patients to different techniques. In fact, our patient collective represents a subsequent evolution of different approaches. However, the comparison showed no relevant differences despite lower PSM for rsRARP. PSM for rsRARP is under debate. In contrast to our results, different authors postulate higher PSM in rsRARP in the primary setting [36,37]. Bahout et al. further evaluated location of PSM and postulate that more PSM could be located at the anterior surface, the bladder neck, and the apex [21]. Comparison to our own data has its limitations as our collective evaluates salvage surgery and not the primary setting. Location of PSM in our rsRARP collective was either at the prostatic apex, the base, or multifocal for all PSM and only multifocal for clinically significant PSM. In addition, the one patient with multifocal PSM and pT2 disease had previously undergone radiation and salvage HIFU before rsRARP.

The impact of PSM on recurrence-free survival and overall survival is also still under debate. While some authors postulate that PSM is associated with higher rate of biochemical recurrence [38], a recent publication showed that cancer specific survival is not affected at all [39]. In addition, evaluation of positive surgical margins shows great variation between different publications. As stated by Preston et al. [40], varying definition of positive surgical margins and variable pathologic specimen handling and reporting may affect PSM results. Therefore, reporting of biochemical recurrence seems more reliable.

Considering oncological outcome, 5-year recurrence free survival rates of 34% to 83% for salvage prostatectomy are described [41]. In our own series, long term follow-up especially for rsRARP is missing. Recurrence rates after 1 year did not favor any of the three presented techniques. Nevertheless, salvage prostatectomy represents a potential curative option for some patients and should therefore not be omitted in patient counselling. Therefore, rsRARP as the latest step in the evolution of salvage prostatectomy represents a valuable and safe surgical option.

## 5. Conclusions

With the adoption of robotic surgery for salvage treatment, the morbidity of the procedure seems to be declining. Considering our results and those from current literature, RARP is a safe and feasible technique also in the salvage situation. Therefore, a minimally invasive procedure should be performed whenever possible. Retzius-sparing RARP as the latest step in the evolution of radical prostatectomy can have advantages, providing direct vision on the anterior rectal wall as the structure of main risk during surgery, especially in the salvage setting. Our data show that this technique, which is exclusive to robotic surgery, is safe and feasible for local recurrence after primary treatment. However, despite advantages of robotic surgery, limits in functional outcome have to be taken into consideration when counselling patients. Oncological outcome, especially for rsRARP, needs longer follow-up for reliable results. A direct comparison of different techniques is not possible as long as prospective randomized clinical trials are pending.

Until then, the choice between cRARP and rsRARP is up to the surgeon’s preference and confidence. Our data were too limited to show significant differences between either technique. Each surgeon has to choose the approach that he is confident with—especially in the salvage setting.

## Figures and Tables

**Table 1:** Patient characteristics.

		All	RRP	cRARP	rsRARP	*p*-Value
*n* (%)		53 (100)	25 (47.2)	7 (13.2)	21 (39.6)	
Age at salvage prostatectomy (years)						0.40
	Mean (sd)	67.6 (5.9)	68.9 (5.8)	66.0 (6.9)	66.5 (5.8)	
	Median (IQR)	68 (64.0–72)	70 (64–74)	69 (61–70)	67 (65–71)	
Initial PSA at diagnosis ng/mL						0.05
	Mean (sd)	12.1 (13.7)	12.2 (13.4)	18.6 (20.9)	9.9 (10.9)	
	Median (IQR)	7.2 (5.2–12.2)	8.5 (6.3–12.3)	12.0 (5.2–21.0)	5.4 (4.5–9.2)	
Initial Gleason score *n* (%)						0.01
	Gleason ≤ 5	6 (11.3)	3 (12)	3 (42.9)		
	Gleason 3 + 3	16 (30.2)	4 (16)	1 (14.3)	11 (52.4)	
	Gleason 3 + 4	10 (18.9)	6 (24)	0	4 (19.0)	
	Gleason 4 + 3	13 (24.5)	6 (24)	3 (42.9)	4 (19.0)	
	Gleason ≥ 8	5 (6.5)	4 (16)	0	1 (4.8)	
	Missing	3 (5.7)	2 (8)	0	1 (4.8)	
Prior treatment *n* (%)						0.12
	Irradiation	27 (50.9)	18 (72)	3 (42.9)	6 (28.6)	
	HIFU	14 (26.4)	4 (16)	1 (14.3)	9 (42.9)	
	IRE	1 (1.9)	0	0	1 (4.8)	
	HDR brachy	1 (1.9)	0	0	1 (4.8)	
	LDR brachy	6 (11.3)	3 (12)	2 (28.6)	1 (4.8)	
	C12 ion irradiation	3 (5.7)	0	1 (14.3)	2 (7.5)	
	Cryoablation	1 (1.9)	0	0	1 (4.8)	
Time between initial and salvage treatment (months)						0.85
	Mean (sd)	52.8 (33.5)	53.0 (33.3)	52.4 (43.8)	52.3 (31.8)	
	Median (IQR)	55 (19–74)	57 (21–77)	51 (17–60)	55 (19–77)	
Clinical stage before salvage RP						0.81
Recurrence cT *n* (%)	1	14 (26.4)	8 (32)	2 (28.6)	4 (19.0)	
	2	30 (56.6)	12 (48)	5 (71.4)	13 (61.9)	
	3 a	5 (9.4)	3 (12)	0	2 (9.5)	
	3 b	3 (5.7)	1 (4)	0	2 (9.5)	
	4	1 (1.9)	1 (4)	0		
PSA before sRP ng/mL						0.01
	Mean (sd)	4.7 (3.9)	6.3 (4.8)	4.3 (2.4)	3.0 (1.9)	
	Median (IQR)	4.1 (2.3–5.7)	5.0 (3.2–7.3)	5.6 (1.8–6.1)	2.5 (1.6–4.4)	
Prostate volume pre-op mL						0.32
	Mean (sd)	23.5 (13.2)	23.2 (8.7)	26.7 (12.0)	22.9 (17.9)	
	Median (IQR)	20.0 (14.0–30.0)	22.0 (17.5–30.0)	25.0 (15.8–36.3)	18.0 (13.3–25.3)	
Perioperative ADT *n* (%)						0.74
	Yes	14 (26.4)	7 (28)	1 (14.3)	6 (28.6)	
	No	39 (73.6)	18 (72)	6 (85.7)	15 (71.4)	
Body mass index						0.69
	Mean (sd)	27.5 (3.2)	27.6 (3.1)	28.0 (4.6)	27.1 (3.0)	
	Median (IQR)	27 (26–29)	27 (26–29)	30 (24–31)	27 (25–29)	

Patient characteristics for patients undergoing salvage radical prostatectomy (sRP), showing age at operation, initial PSA (ng/mL), initial Gleason score, prior treatment, time between initial treatment and salvage surgery, clinical T-stage at time of recurrence, PSA before surgery (ng/mL), prostate volume (mL), and perioperative androgen deprivation therapy (ADT) for all study groups and separately for retropubic open (RRP), conventional (cRARP) and Retzius-sparing (rsRARP) robotic assisted radical prostatectomy.

**Table 2:** Peri- and post-operative parameters.

		All	RRP	cRARP	rsRARP
*n* (%)		53 (100%)	25 (47.2)	7 (13.2)	21 (39.6)
Blood loss mL	Median (IQR)	500 (300–950)	900 (550–1350)	500 (300–600)	300 (200–500)
Operation time min	Median (IQR)	215 (191–247)	205 (176–225)	252 (210–330)	228 (206–259)
Pathological T Stage (%)					
	1	0	0	0	0
	2	27 (50.9%)	9 (36.0%)	3 (42.9%)	15 (71.4%)
	3	25 (47.2%)	15 (60.0%)	4 (57.1%)	6 (28.6%)
	4	1 (1.9%)	1 (4.0%)	0	0
Immediate continence					
Continent		5 (9.4)	2 (8.0)	0	3 (14.3)
Stress incontinence					
	Grade 1	11 (20.8)	7 (28.0)	1 (14.3)	3 (14.3)
	Grade 2	16 (30.2)	8 (32.0)	4 (57.1)	4 (19.0)
	Grade 3	14 (26.4)	8 (32.0)	1 (14.3)	5 (23.8)
Missing		7 (13.2)	0	1 (14.3)	6 (28.6)
Continence at 12 months post sRP *n* (%)					
Continent		9 (17.0%)	5 (20.0%)	0	4 (19.0%)
Stress incontinence					
	Grade 1	19 (35.8%)	9 (36.0%)	3 (42.9%)	7 (33.3%)
	Grade 2	10 (18.9%)	5 (20.0%)	2 (28.6%)	3 (14.3%)
	Grade 3	9 (17.0%)	3 (12.0%)	2 (28.6%)	4 (19.0%)
Missing		6 (11.3%)	3 (12.0%)		3 (14.3%)
Hospital stay (days)					
	Mean (sd)	7.7 (4.2)	9.9 (4.7)	5.4 (1.3)	5.7 (2.6)
	Median (IQR)	7.0 (5.0–9.0)	9.0 (7.5–10.5)	5.0 (4.0–7.0)	5.0 (4.0–6.5)
Surgical margins					
R0		33 (62.3%)	15 (60.0%)	2 (28.6%)	16 (76.2%)
R1		17 (32.1%)	9 (36.0%)	4 (57.1%)	4 (19.0%)
R2		1 (1.9%)	1 (4.0%)	0	0
RX		2 (3.8%)	0	1 (14.3%)	1 (4.8%)
Catheterization (days)					
	Mean (sd)	35.7 (28.0)	41.7 (26.4)	47.6 (41.4)	24.5 (21.5)
	Median (IQR)	28.0 (15.5–42.0)	37.0 (27.0–51.5)	27.0 (20–96)	20.0 (13.0–34.0)

Peri- and postoperative characteristics blood loss (mL), operation time (min), pathological T-stage, immediate continence after catheter removal, continence at 12 months after surgery, patient age at time of operation, duration of hospital stay (days), surgical margins (R0: no cancer cells seen microscopically at tumour site; R1: cancer cells present microscopically at the primary tumour site; R2: macroscopic residual tumour at primary cancer site; Rx: cannot be determined), initial PSA at diagnosis (ng/mL), BMI, and duration for indwelling catheter (days) for all study groups and separately for retropubic open (RRP), conventional and Retzius-sparing robotic assisted radical prostatectomy (cRARP/rsRARP).

## Data Availability

The data presented in this study are available on request from the corresponding author. The data are not publicly available due to ethical restrictions.
